# Post-traumatic stress in older, community-dwelling adults with hypertension during the COVID-19 pandemic: An investigation of pre-pandemic sociodemographic, health, and vascular and inflammatory biomarker predictors

**DOI:** 10.1177/13591053231213305

**Published:** 2023-12-13

**Authors:** Emily A Troyer, Jordan N Kohn, Monica Feliz R Castillo, Judith D Lobo, Yaniel Ramirez Sanchez, Gavrila Ang, Anthony Cirilo, Juan Andrew Leal, Christopher Pruitt, Amanda L Walker, Kathleen L Wilson, Meredith A Pung, Laura S Redwine, Suzi Hong

**Affiliations:** 1 University of California, USA; 2 University of Miami, USA

**Keywords:** aging, biomarkers, COVID-19, hypertension, post-traumatic stress

## Abstract

COVID-19 pandemic-related traumatic stress (PRTS) symptoms are reported in various populations, but risk factors in older adults with chronic medical conditions, remain understudied. We therefore examined correlates and pre-pandemic predictors of PRTS in older adults with hypertension during COVID-19. Participants in California, aged 61–92 years (*n* = 95), participated in a pre-pandemic healthy aging trial and later completed a COVID-19 assessment (May to September 2020). Those experiencing ⩾1 PRTS symptom (*n* = 40), and those without PRTS symptoms (*n* = 55), were compared. The PRTS+ group had poorer mental and general health and greater impairment in instrumental activities of daily living. Pre-pandemic biomarkers of vascular inflammation did not predict increased odds of PRTS; however, greater pre-pandemic anxiety and female gender did predict PRTS during COVID-19. Our findings highlight PRTS as a threat to healthy aging in older adults with hypertension; targeted approaches are needed to mitigate this burden, particularly for females and those with pre-existing anxiety.

Post-traumatic stress disorder (PTSD) is characterized by persistent mental and emotional distress lasting at least 1 month following a traumatic event, clinically defined by post-traumatic stress symptoms, which include (i) intrusion phenomena, (ii) avoidance, (iii) hypervigilance, and (iv) negative alterations in mood and cognition (APA, 2013). The coronavirus pandemic is a unique shared stressor, particularly for adults over 65 years of age with chronic medical conditions, who are at greater risk of hospitalization and death due to COVID-19, compared to younger adults (Taylor et al., 2023). Prior to the pandemic, the past-year prevalence of PTSD in the United States was 4.7% (Goldstein et al., 2016), and meta-analyses from studies during the pandemic have shown community prevalence of PTSD in several countries ranging from 15% to 23% (Cénat et al., 2021; Cooke et al., 2020; Krishnamoorthy et al., 2020).

PTSD is associated with increased incidence of various comorbidities, particularly cardiovascular disease (CVD) due to the well-characterized effects of stress on cardiovascular risk. For example, PTSD is linked to incident hypertension (HTN), acute cardiac events, stroke, and CVD-related mortality (Howard et al., 2018; Wentworth et al., 2013; Xue et al., 2012), which can be explained by both behavioral (e.g. sleep difficulties, alcohol and tobacco use, physical inactivity) and physiological mechanisms (e.g. autonomic imbalance, hypothalamic-pituitary-adrenal axis activation, inflammation) (Scherrer et al., 2019). Individuals with PTSD, or chronic psychological distress and traumatic life events more generally, exhibit higher levels of blood proinflammatory biomarkers such as interleukin (IL)-1β, IL-6, tumor necrosis factor (TNF)-α, and C-reactive protein (CRP), compared to healthy controls (Knight et al., 2021; Speer et al., 2018). Interestingly, these same biomarkers are also implicated in HTN, CVD pathogenesis, and aging (Dinh et al., 2014). Therefore, inflammatory dysregulation might be a shared pathogenic mechanism underlying associations between traumatic stress and adverse health outcomes, particularly in older aged adults. PTSD also confers risk for age-related cognitive deficits and neurodegenerative disorders including frontotemporal and vascular dementias, and Alzheimer’s disease (Qureshi et al., 2010; Sperling et al., 2011; Yaffe et al., 2010). Furthermore, elevated pre-trauma inflammatory biomarker levels predict later risk of developing PTSD (Eraly et al., 2014; Sumner et al., 2018), and whether this extends to elevated risk of developing subclinical PTSD symptoms following exposure to a shared traumatic event, such as the pandemic, is unknown. Taken together, studies suggest that traumatic stress impacts vascular diseases (e.g. stroke, myocardial infarction) that have increased prevalence in older age, likely via complex multifactorial biopsychosocial mechanisms. However, the relationship between subclinical PTSD symptoms, specifically pandemic-related traumatic stress symptoms (PRTS), and physical and mental health in older individuals are less well understood, particularly among those with pre-existing CVD risk factors such as HTN. Therefore, disentangling these complex processes is important for characterizing and mitigating the traumatic stress-associated burden of the COVID-19 pandemic.

PRTS during the pandemic in older adults with CVD risk factors associated with chronic inflammation remains underexplored; therefore, we aimed to investigate correlates and predictors of PRTS in older adults with HTN in a well-characterized cohort previously enrolled in a longitudinal behavioral intervention trial. We assessed PRTS and other aspects of health and psychosocial functioning after onset of the pandemic in older adults, for whom pre-pandemic sociodemographic, general, mental, cognitive, and cardiovascular health, and vascular inflammatory biomarker data were also available. First, we hypothesized that (i) older adults with HTN who reported PRTS during the COVID-19 pandemic would have poorer self-rated general and mental health, poorer sleep quality, less adaptive functioning, and have worse cognitive status as compared to participants who did not report PRTS. We further hypothesized that (ii) pre-pandemic biopsychosocial factors would confer increased risk of PRTS after onset of the pandemic, including baseline sociodemographic characteristics, measures of cardiovascular and mental health, and vascular inflammatory biomarkers.

## Methods

### Participants

Individuals aged 60 years or older with HTN who participated (May 2016 to July 2020) or volunteered to participate in a larger healthy aging trial (Healthy Aging Practice-centered Instruction Cardiovascular Health Investigation, HAPI-CHI) were invited to participate in the current COVID-19 extension study in San Diego County, California. The HAPI-CHI Study aimed to investigate cardiovascular, immune, and psychological factors as they related to blood pressure changes in response to 12-weeks of Tai Chi (TC) versus Healthy Aging Practice-centered Education (HAP-E). For the parent study, a diagnosis of HTN was a key inclusion criterion (systolic blood pressure, SBP = 130–170 mmHg), and use of antihypertensive medication(s) was allowed. Key exclusion criteria included inability to perform light to moderate exercise; regular moderate exercise or ongoing meditation practice; body mass index (BMI) >50 kg/m^2^; past-year stroke or myocardial infarction (MI), or fall requiring hospitalization; oxygen-dependent chronic obstructive pulmonary disease, chronic kidney disease, or malignancy; serious mental illness which could interfere with study participation; current major depressive episode or suicidality; and current use of mood stabilizing, antipsychotic, or immune-modulating medications (e.g. systemic corticosteroids). Comprehensive inclusion and exclusion criteria for HAPI-CHI have also been described elsewhere (Kohn et al., 2020, 2023a, 2023b).

For the parent trial (HAPI-CHI) from 2016 to 2020, 182 participants were randomly assigned to a 12-week health behavioral intervention arm (TC *n* = 97 or HAP-E *n* = 85), and 166 participants attended at least one class and a pre-COVID study visit. We attempted to re-contact individuals who had previously consented to being contacted for follow-up/future studies (*n* = 137) for participation in the COVID-19 assessment. Of those, 112 were successfully re-contacted, and 95 participants completed the COVID-19 survey.

### Procedures

For the present study, participants were contacted in April 2020 and invited to participate in a survey to assess the impact of the pandemic, administered either online via Qualtrics software, or via a mailed paper survey, per participant preference. Ninety-five participants, 61–92 years of age, completed the survey from May to September 2020. At this time in the COVID-19 pandemic, vaccines and monoclonal antibodies against SARS-CoV-2 were not yet developed, the State of California had declared a state of emergency (March 2, 2020) and enacted a statewide stay-at-home order (March 19, 2020) that persisted throughout the survey period, and the US had recorded over 1 million SARS-CoV-2 infections (April 28, 2020). Assessments immediately after completion of the health behavioral intervention are referred to as “pre-COVID” assessments, and surveys administered during the pandemic as “COVID-19” assessments. Procedures and measures administered at both timepoints are detailed in the following sections and summarized in Table 1. Additionally, time between study visits (pre-COVID and COVID-19), and time between the California state of emergency declaration and the date of COVID-19 survey completion were calculated for each participant.

**Table 1.** Overview of procedures and measures administered at each assessment timepoint.

	Pre-COVID assessment	COVID-19 assessment
*Sociodemographic factors*		
Age, gender, race, marital status, first language, education	+	**−**
*Pandemic-related traumatic stress symptoms*		
PC-PTSD	**−**	+
*COVID-19 pandemic-related stressors*		
CRISIS questionnaire	**−**	+
*General health*		
Medical diagnoses	+	**−**
Tobacco, cannabis, and alcohol use	+	+
SF-20	+	+
PROMIS-SD	+	+
SBQ	**−**	+
*Mental health*		
BDI-II	+	+
PROMIS-A	+	+
CD-RISC-10	+	+
GQ-6	+	+
ULS-8	**−**	+
SCS-SF	**−**	+
*Cognitive status*		
MoCA	+	**−**
FAQ	**−**	+
*Cardiovascular health*		
Anti-hypertensive medications	+	+
Mean SBP and DBP	+	**−**
Lipids, CBC	+	**−**
Inflammatory and vascular injury biomarkers	+	**−**

(+) Domain was assessed at this timepoint. (−) Domain was not assessed at this timepoint.

PC-PTSD: Primary Care PTSD Screen; CRISIS: CoRonavIruS health and Impact Survey; SF-20: 20-Item Short Form Health Survey; PROMIS: Patient-Reported Outcomes Measurement Information System; PROMIS-SD: PROMIS Sleep Disturbance, Adult Short Form 8a; SBQ: Sedentary Behavior Questionnaire; BDI-II: Beck Depression Inventory, Second Edition; PROMIS-A: PROMIS Anxiety, Adult Short Form 8a; CD-RISC-10: 10-Item Connor-Davidson Resilience Scale; GQ-6: Gratitude Questionnaire 6-Item Form; ULS-8: 8-Item UCLA Loneliness Scale; SCS-SF: Self Compassion Scale – Short Form; MoCA: Montreal Cognitive Assessment, Version 7; FAQ: Functional Activities Questionnaire; SBP: systolic blood pressure; DBP: diastolic blood pressure; CBC: complete blood count.

This study was approved by the Institutional Review Board at the University of California, San Diego. Upon study enrollment, participants provided written informed consent and demonstrated sufficient understanding of the study via the Brief Assessment of Capacity to Consent (Jeste et al., 2007).

#### Sociodemographic factors

Sociodemographic information was collected at the pre-COVID assessment, during in-person study visits (age in years, gender, race, marital status, first language, educational attainment).

#### Pandemic-related stressors and traumatic stress symptoms

COVID-19 pandemic-related stressors were assessed during COVID-19 using an adapted version of the CoRonavIruS health and Impact Survey (CRISIS), which was designed to assess the impact of the pandemic in various domains (Nikolaidis et al., 2021).

The primary outcome of interest, PRTS, was assessed during COVID-19, using the Primary Care PTSD Screen (PC-PTSD) (Prins et al., 2004), which assesses past-month presence of re-experiencing, avoidance, hyperarousal, and numbing. Scores range from 0 to 4, depending on number of items endorsed. Using a cut-off score of ⩾3, the PC-PTSD has 78% sensitivity, 87% specificity, and 85% efficiency for a clinical diagnosis of PTSD (Prins et al., 2004). In this study, participants were instructed to respond to items with reference to the pandemic (i.e. “In the past month, have you experienced any of the following symptoms related to the COVID-19 pandemic?”). The term PRTS, rather than PTSD, is used here, to emphasize that participants were not clinically diagnosed with PTSD.

#### General health and behaviors

##### Pre-COVID

Information about general health and behavior, including medical diagnoses and prescription medications, were ascertained via semi-structured in-person interviews at the pre-COVID assessment.

##### Pre-COVID and during COVID-19

Self-reported use of tobacco, cannabis, and alcohol were recorded at both timepoints. The Medical Outcomes Study (MOS) 20-Item Short Form Health Survey (SF-20) (Stewart and Ware, 1992) and the Patient-Reported Outcomes Measurement Information System (PROMIS) Sleep Disturbance scale, Adult Short Form 8a (PROMIS-SD) (Yu et al., 2012) were administered pre-COVID and during COVID-19, to assess domains of general health and sleep disturbances, respectively.

##### During COVID-19

The Sedentary Behavior Questionnaire (SBQ) was administered during COVID-19 to quantify time spent engaged in nine sedentary behaviors on a typical day during the pandemic (Rosenberg et al., 2010).

#### Mental health

##### Pre-COVID and during COVID-19

Mental health was assessed at both timepoints. Self-report measures of depressive and anxious symptoms included the BDI-II (Beck et al., 1996) and the PROMIS Anxiety (PROMIS-A), specifically Adult Short Form 8a (Pilkonis et al., 2011). Resilience and gratitude were assessed at both timepoints, using the 10-Item Connor-Davidson Resilience Scale (CD-RISC-10) (Connor and Davidson, 2003), and the Gratitude Questionnaire 6-Item Form (GQ-6) (McCullough et al., 2002), respectively.

##### During COVID-19

Measures administered only during COVID-19 included the 8-Item UCLA Loneliness Scale (ULS-8) (Hays and DiMatteo, 1987) and the Self Compassion Scale—Short Form (SCS-SF) (Raes et al., 2011).

#### Cognitive status

##### Pre-COVID

Cognitive status was assessed pre-COVID, using one of three alternate forms of Version 7 of the full Montreal Cognitive Assessment (MoCA), a brief screening measure for cognitive impairment (Nasreddine et al., 2005). The MoCA was administered in a randomized, counterbalanced order by trained research personnel.

##### During COVID-19

Cognition was not objectively assessed during the pandemic, given the survey format. However, the Functional Activities Questionnaire (FAQ) (Pfeffer et al., 1982) was administered, which measures impairment in instrumental activities of daily living (IADLs), and importantly, ability to independently perform IADLs is related to cognitive status, particularly executive function (Mlinac and Feng, 2016).

#### Cardiovascular health

##### Pre-COVID

Mean systolic and diastolic blood pressures (SBP, DBP) were calculated based on three consecutive pre-pandemic measurements using an automated oscillometric sphygmomanometer. A 10-year cardiovascular Framingham Risk Score (FRS) (Lloyd-Jones et al., 2004) was also calculated using baseline sociodemographic, medical history, and laboratory data.

##### Pre-COVID and during COVID-19

Current medications were recorded at both assessments, and anti-HTN medications were categorized into one of eight classes. For each participant, the sum of unique anti-HTN medication classes prescribed was recorded. Given the high proportion of medication use, each participant’s sum of unique anti-HTN medications (74.4% taking ⩾1; mean = 1.31, SD = 1.15) was used as a proxy for HTN severity.

#### Inflammatory and vascular injury biomarkers

Blood samples were collected at the pre-COVID assessment, at a similar time, after at least 12 hours of abstinence from anti-inflammatory medications, caffeine, nicotine, and strenuous exercise, into sterile EDTA tubes. Plasma was collected using a refrigerated centrifuge and stored at −80°C for subsequent vascular and inflammatory biomarker quantification. Whole blood samples were sent to a CLIA-certified laboratory (LabCorp, San Diego, CA) for evaluation of complete blood counts and lipid profiles, to rule out infection and to calculate FRSs, respectively.

Inflammatory and vascular injury markers, including CRP, serum amyloid A (SAA), soluble vascular cell adhesion molecule-1 (sVCAM-1), soluble intercellular adhesion molecule-1 (sICAM-1), IL-6, and TNF-α were examined given their putative associations with PTSD, HTN, and cardiovascular disease (Dinh et al., 2014; Eraly et al., 2014; Speer et al., 2018; Sumner et al., 2018). Concentrations were measured using electrochemi-luminescence-based multi-array sandwich immunoassays, 96-well-based high throughput platforms. Levels of CRP, SAA, sVCAM-1, and sICAM-1 were measured using the Meso Scale Discovery (MSD) Vascular Injury Panel-2 V-PLEX (human) 4-spot multiplex kit, and IL-6 and TNF-α using the MSD Human Proinflammatory Panel-1 V-PLEX (human) 10-spot multiplex kit (Meso Scale Diagnostics LLC, Rockville, MD, USA). Inter-assay and intra-assay coefficients of variation (CVs) averaged <10%. Lower limits of detection (LLOD) and original concentration units for analytes were as follows: CRP = 1.33 pg/mL, SAA = 10.9 pg/mL, sVCAM-1 = 6.00 pg/mL, sICAM-1 = 1.03 pg/mL, IL-6 = 0.06 pg/mL, and TNF-α = 0.04 pg/mL.

### Statistical methods

Statistical analyses were conducted in R (v.3.6.0). All variables were inspected for normality and outliers. Participants with PC-PTSD scores ⩾1 were classified as “PRTS+,” and participants with PC-PTSD scores = 0 were classified as “PRTS−.” Group comparisons were conducted using nonparametric, rank-biserial correlation coefficients (*r*_rb_) for non-normal continuous variables (Kerby, 2014). Welch’s *t*-test for normally distributed continuous variables, and categorical variables were compared using Chi-squared (χ^2^) or Fisher’s exact tests. Missingness was <5% for all regression predictors, except FRS, which had ~20% missingness. Multiple imputation by chained equations (*mice*) (Azur et al., 2011), using predictive mean matching with 100 imputations was implemented to account for missing data in logistic regression analyses. Binomial logistic regression was applied to each of the imputed datasets, and results were pooled to determine odds ratios (*ORs*) and 95% confidence intervals for each baseline variable in predicting “PRTS+” status during the pandemic. Likelihood ratio tests were computed from pooled models using the *D3-*statistic (Meng and Rubin, 1992) to compare a reduced model containing age, gender, number of anti-HTN medications, FRS, and inflammatory and vascular injury biomarkers, to a full model that also contained depressive symptoms, anxiety, sleep disturbance, and MoCA scores.

Dimensionality reduction by principal components analysis (PCA) was performed using *principal* in *psych* for the six inflammatory and vascular injury biomarkers due to their high multicollinearity (mean Spearman’s correlation (*r*_s_) = 0.36, range = 0.10–0.72, SD = 0.19) to extract component scores for each participant (see Supplemental Figure 1, a correlation matrix of the six pre-COVID biomarkers). Complete biomarker data were available for 80 of 95 (84%) of participants, and missing values were imputed using an iterative, regularized PCA algorithm in the *missMDA* package. Imputed biomarker data were scaled and standardized prior to PCA, for which the first component (PC1) accounted for 48% of the total variance and upon which all biomarkers loaded stably (standardized loadings ⩾0.60), except for TNF-α (0.30). Varimax-rotated component scores from PC1 were used as the inflammatory and vascular injury biomarker index in logistic regression analysis, and correlations between PC1 and other variables were described. False discovery rate (FDR) correction was applied to biomarker correlations to control Type I error rate due to multiple comparisons.

## Results

### COVID-19 survey respondents versus non-respondents

Of those invited to participate, COVID-19 survey respondents were younger, more likely to be married, and showed higher pre-COVID MoCA scores compared to COVID-19 survey non-respondents. The groups did not differ in other pre-COVID factors (see Supplemental Tables 1 and 2, which show comparisons of pre-pandemic sociodemographic characteristics and health measures for COVID-19 assessment respondents vs non-respondents).

### PRTS at the COVID-19 assessment

Of 95 COVID-19 survey respondents, 40 (42%; 95% CI: 32%–52%) endorsed one or more symptoms on the PC-PTSD (i.e. “PRTS+”), and the remaining 55 participants (58%) reported no such symptoms (i.e. “PRTS−”). A threshold score of 1 on the PC-PTSD was used for group comparisons to capture any perceived traumatic stress during the pandemic, rather than a probable clinical diagnosis of PTSD. Nevertheless, 7 of 95 (7.4%) survey respondents endorsed three or more symptoms on the PC-PTSD, the cut-off score with optimal sensitivity, specificity, and efficiency for probable PTSD (Prins et al., 2004).

### Biopsychosocial factors by PRTS status at the COVID-19 assessment

The PRTS+ group was disproportionately female, but the groups did not otherwise differ in pre-pandemic characteristics, including age, race, marital status, first language, educational attainment, or HAPI-CHI study arm (see Table 2). Time elapsed between the pre-COVID and COVID-19 assessments did not differ by PRTS group. However, time since onset of California’s state of emergency (March 2, 2020) to completion of the COVID-19 assessment was significantly longer in the PRTS+ group compared to the PRTS− group (142.7 ± 36.0 vs 125.2 ± 32.8 days, χ^2^ = 5.64, *p* < 0.05) (see Supplemental Table 3). Types of pandemic-specific stressors on the CRISIS questionnaire did not differ between groups, and no participants reported having tested positive for COVID-19 infection (see Supplemental Table 4, which compares CRISIS questionnaire responses by self-reported PRTS).

**Table 2.** Sociodemographic characteristics at the pre-COVID assessment by PRTS group.

	PRTS+ (*n* = 40)	PRTS− (*n* = 55)	Test statistic [95% CI]
Age: mean (SD)	74.15 (8.24)	73.94 (6.12)	*t*_67_ = −0.28 [−3.44, 2.59]
Gender: % female	33/40 (82.5)	31/55 (56.4)	χ^2^ = 6.06*
Race: % Caucasian	31/37 (83.8)	43/53 (79.3)	χ^2^ = 0.002
Marital status: % married	17/40 (42.5)	22/55 (40.0)	χ^2^ = 0.001
First language: % English	33/40 (82.5)	50/55 (90.9)	χ^2^ = 0.82
Education: % some college or greater	34/40 (85.0)	45/54 (83.3)	χ^2^ = 0.000
Study arm: % assigned to Tai Chi	14/40 (35.0)	31/55 (56.4)	χ^2^ = 3.43

PRTS: pandemic-related traumatic stress symptoms; CI: confidence interval; SD: standard deviation; χ^2^: Chi-squared.

* *p* < 0.05.

Regarding general health and behavior, the groups did not differ in self-reported tobacco, alcohol, or cannabis use. PRTS+ group participants reported worse perception of their general health and physical functioning, along with greater levels of pain, and greater sleep disturbances, compared to those without PRTS. There were no group differences in self-reported social or role functioning, nor were there differences in self-reported sedentary behavior (see Table 3).

**Table 3.** General, mental, and cognitive health self-report measures at the COVID-19 assessment based on self-reported PRTS.

	PRTS+ (*n* = 40)	PRTS− (*n* = 55)	Test statistic [95% CI]
Smoking: % using tobacco	0/40 (0.00)	1/54 (1.85)	OR = 0.00 [0.00, 52.6]
Cannabis: % any cannabis use	1/40 (2.50)	3/55 (5.45)	OR = 0.45 [0.01, 5.83]
Alcohol: % any alcohol use	19/40 (47.5)	31/55 (56.4)	χ^2^ = 0.418
SF-20 social functioning: mean (SD)	78.5 (32.2)	85.8 (28.2)	*r*_rb_ = 0.14 [−0.10, 0.36]
SF-20 pain: mean (SD)	53.5 (23.3)	67.3 (22.9)	*r*_rb_ = 0.33 [0.10, 0.52]**
SF-20 health perception: mean (SD)	49.6 (20.2)	58.3 (20.1)	*r*_rb_ = 0.30 [0.08, 0.50]*
SF-20 role functioning: mean (SD)	68.1 (43.1)	79.6 (37.9)	*r*_rb_ = 0.14 [−0.09, 0.36]
SF-20 physical functioning: mean (SD)	59.8 (28.6)	77.0 (27.0)	*r*_rb_ = 0.38 [0.16, 0.56]**
PROMIS-SD: mean (SD)	52.2 (7.7)	44.8 (6.8)	*t*_76_ = 4.84 [4.36, 10.4]***
SBQ: mean (SD)	65.8 (20.4)	66.3 (25.9)	*t*_93_ = 0.11 [−8.92, 9.95]
SF-20 mental health: mean (SD)	71.9 (17.1)	85.8 (13.6)	*r*_rb_ = 0.51 [0.31, 0.66]***
BDI-II: mean (SD)	10.18 (6.26)	4.22 (4.09)	*r*_rb_ = −0.59 [−0.72, −0.42]***
PROMIS-A: mean (SD)	55.1 (7.0)	46.0 (6.6)	*t*_81_ = −6.37 [−11.9, −6.25]***
ULS-8: mean (SD)	53.5 (16.1)	41.52 (12.1)	*t*_69_ = −3.96 [−18.0, −5.96]***
CD-RISC-10: mean (SD)	27.1 (8.26)	31.4 (5.77)	*r*_rb_ = 0.31 [0.08, 0.51]*
SCS-SF: mean (SD)	41.4 (9.10)	47.3 (7.74)	*r*_rb_ = 0.38 [0.16, 0.56]**
GQ-6: mean (SD)	35.7 (5.15)	36.8 (5.19)	*r*_rb_ = 0.16 [−0.08, −0.37]
FAQ: mean (SD)	2.50 (3.51)	1.12 (2.15)	*r*_rb_ = −0.29 [−0.49, −0.06]**

PRTS: pandemic-related traumatic stress symptoms; CI: confidence interval; SF-20: 20-Item Short Form Health Survey; SD: standard deviation; PROMIS: Patient-Reported Outcomes Measurement Information System; PROMIS-SD: PROMIS Sleep Disturbance, Adult Short Form 8a; SBQ: Sedentary Behavior Questionnaire; BDI-II: Beck Depression Inventory, Second Edition; PROMIS-A: PROMIS Anxiety, Adult Short Form 8a; ULS-8: 8-Item UCLA Loneliness Scale; CD-RISC-10: 10-Item Connor-Davidson Resilience Scale; SCS-SF: Self-Compassion Scale—Short Form; GQ-6: Gratitude Questionnaire 6-Item Form; FAQ: Functional Activities Questionnaire; OR: odds ratio (based on Fisher’s Exact Test); χ^2^: Chi-squared; *r*_rb_: rank biserial correlation coefficient.

* *p* < 0.05. **p < 0.01. ***p < 0.001.

The PRTS+ group reported significantly worse perception of their overall mental health on the SF-20, greater depressive symptoms on the BDI-II, and higher anxiety on the PROMIS-A. The PRTS+ group also self-reported greater loneliness, less resilience, and less self-compassion, but no difference in gratitude, relative to the PRTS− group (see Table 3).

In terms of cognitive status, the PRTS+ group self-reported significantly greater impairment in IADLs on the FAQ, compared to the PRTS− group (see Table 3).

We performed a sensitivity analysis wherein we repeated comparisons of sociodemographic and biopsychosocial factors between PRTS groups defined using a PC-PTSD cut-off score of ⩾3 (see Supplemental Tables 5 and 6). In both analyses (using either a cut-off score of ⩾1 or a cut-off score of ⩾3), the PRTS+ group had worse perception of their general health, greater levels of pain, greater sleep disturbances, worse overall mental health, higher depressive and anxiety symptoms, less resilience and self-compassion, and significantly greater impairment in IADLs (see Table 3 and Supplemental Table 6).

### Pre-COVID predictors of PRTS during the COVID-19 pandemic

First, sociodemographic factors, cardiovascular health, and vascular and inflammatory biomarkers were considered as predictors of PRTS status. Pre-COVID, PRTS+, and PRTS− groups did not differ in age, anti-HTN medication usage, FRS, or inflammatory biomarker levels. FRS was marginally correlated with inflammatory biomarkers (*r*_s_ = 0.21, *p* = 0.07) and MoCA scores (*r_s_* = −0.19, *p* = 0.09; see Supplemental Figure 2). Despite ORs > 1 for anti-HTN medications, FRS, and inflammation index, logistic regression revealed that the pre-COVID cardiovascular health and biomarker factors examined did not significantly predict increased COVID-19-related PRTS. Of note, women had significantly greater odds of PRTS+ status compared to men (see Table 4).

**Table 4.** Logistic regression models of pre-COVID-19 predictors of PRTS status during the COVID-19 pandemic.

	Pre-COVID predictor variables	OR	*df*	95% CI	
				Lower	Upper
Model 1	Gender	3.968	83.748	1.281	12.294
	Age in years	0.995	85.798	0.931	1.064
	Anti-hypertensive medications	1.031	83.119	0.669	1.59
	FRS	1.955	67.013	0.003	1422
	Inflammation index	1.211	86.857	0.925	1.587
Model 2	Gender	5.508	76.191	1.417	21.416
	Age in years	0.982	79.734	0.903	1.069
	Anti-hypertensive medications	1.128	74.762	0.67	1.901
	FRS	0.910	56.755	0.000	4205
	Inflammation index	1.173	81.662	0.827	1.664
	BDI-II scores	0.941	81.393	0.841	1.053
	PROMIS-SD scores	1.057	80.453	0.994	1.124
	PROMIS-A scores	1.135	79.864	1.047	1.231
	MoCA scores	1.142	80.546	0.909	1.434

CI: confidence interval; OR: odds ratio; *df*: degrees of freedom; FRS: Framingham Risk Score; BDI-II: Beck Depression Inventory, Second Edition; PROMIS: Patient-Reported Outcomes Measurement Information System; PROMIS-SD: PROMIS Sleep Disturbance, Adult Short Form 8a; PROMIS-A: PROMIS Anxiety, Adult Short Form 8a; MoCA: Montreal Cognitive Assessment, Version 7.

A subsequent logistic regression model was fit which included the prior predictors, as well as additional general and mental health and cognitive status predictors (i.e. sleep disturbances, depressive and anxious symptoms, and MoCA scores). This full model was a significantly better fit than the aforementioned model (χ^2^ = 4.82, *df* = 4, *p* < 0.001) and demonstrated that female gender and greater pre-pandemic anxiety symptoms were associated with significantly increased risk of PRTS+ status during the pandemic. Pre-COVID anti-HTN medications, inflammation index, sleep disturbance, and MoCA scores were not significant predictors of PRTS+ status (see Table 4).

## Discussion

In this cohort of older adults with HTN during the COVID-19 pandemic, 42% of respondents endorsed at least 1 PRTS symptom. This is consistent with other studies in older adults during the pandemic, where PRTS prevalence is around 36%–38% (Armitage et al., 2022; Jassal et al., 2022). Our finding of potentially clinically elevated PTSD symptoms (PC-PTSD cut-off score ⩾3) in 7.4% of study participants is greater than the pre-pandemic prevalence of PTSD in the general population of 4.7% (Goldstein et al., 2016), but lower than previously reported in meta-analyses of adult samples since onset of the pandemic (ranging 15%–23%) (Cénat et al., 2021; Cooke et al., 2020; Krishnamoorthy et al., 2020). This is consistent with studies suggesting that younger adults report greater pandemic-related disruptions to their lives and higher rates of pandemic-related PTSD compared to older adults (Carney et al., 2021; Rossi et al., 2020). Nevertheless, the frequency with which PRTS progresses into clinically diagnosed PTSD among older adults remains a critical question for future exploration.

Cross-sectionally at the COVID-19 assessment, participants in the PRTS+ group self-reported greater depression, anxiety, and loneliness, and lower resilience and self-compassion, relative to the PRTS− group. This is not surprising, given the frequent comorbidity of trauma-related and other psychiatric symptoms (Kessler et al., 1995). The PRTS+ group also reported poorer general health, decreased physical functioning, greater sleep disturbance, and more subjective impairment in instrumental activities of daily living (IADLs). These findings are consistent with other studies indicating that pandemic-related chronic stress among older adults is associated with poorer sleep (Kuo et al., 2023), and greater physical disability is correlated with larger pandemic-related increases in depressive symptoms (Li and Luo, 2023). Although we did not perform objective assessments of cardiovascular health and cognitive function during the pandemic, our findings suggest that older adults with PRTS experienced poorer subjective general health and cognitive functioning. Indeed, longitudinal analyses indicate that community-dwelling older adults in the US with HTN or CVD experienced accelerated rates of cognitive decline during the COVID-19 pandemic relative to rates observed during pre-pandemic assessment (Hua et al., 2023; Li et al., 2023). Pre-existing cardiovascular risk factors such as HTN, and traumatic stress, are both conceptualized as mechanisms in accelerating biological senescence (Lohr et al., 2015), and the cumulative effect of HTN and PRTS during COVID-19 on long-term risk for CVD-related outcomes (i.e. stroke, myocardial infarction) and cognitive decline, or incident neurocognitive disorders, in older adults remains to be fully elucidated. To date, no studies to our knowledge have examined these outcomes in older adults with HTN and PRTS during COVID-19, so this remains a critical area of inquiry.

Notably, time since onset of California’s state of emergency (March 2, 2020) to completion of the COVID-19 study assessment was longer in the PRTS+ group compared to the PRTS− group, suggesting that greater duration of exposure to the pandemic could be associated with PRTS+ status. However, all assessments were conducted over a 4-month period, from May to September 2020, during which the status of the pandemic was relatively consistent. At the beginning of the study period, the US had already reported over 1 million cases (April 28, 2020), and so the implications of the pandemic (and therefore magnitude of the stressor) had been appreciated by all study participants. Throughout the study period, both groups were exposed to a consistent pattern of mitigation strategies (i.e. California maintained a statewide mask mandate and stay-at-home order, and vaccines and monoclonal antibody treatments for COVID-19 infection were not available). The PRTS groups also reported experiencing similar pandemic-related stressors on the CRISIS questionnaire, and no participants reported having been infected with COVID-19. All participants were invited to complete the COVID-19 assessment at the beginning of the study period, and it could be the case that PRTS+ status, or associated differences in other health domains during the pandemic, contributed to individuals in the PRTS+ status group delaying their participation to later in the study period.

We also found that gender and pre-pandemic anxiety scores significantly predicted PRTS. Female gender as a predictor of PRTS is consistent with previous studies during the pandemic, suggesting that among healthcare workers and the general population, women are at greater risk for PRTS (Liu et al., 2020). In response to traumatic events more generally, women are more likely than men to develop clinical PTSD (Kimerling et al., 2018). Among older adults, specifically, a nationally representative longitudinal sample from England reported increased levels of anxiety during the COVID-19 pandemic (Zaninotto et al., 2022), with women experiencing larger increases than men (i.e. interaction effect) for anxiety and other mental health outcome (e.g. quality of life, loneliness). Similarly, older women in the UK reported greater PRTS than older men (assessed using the Impact of Event Scale) (Armitage et al., 2022). However, the mediators of these women-specific effects remain unclear: greater biologic risk for PRTS in females (e.g. neuroendocrine processes), disproportionate psychosocial impact of the pandemic on women (e.g. caregiving burden, isolation), or a combination of both. Further studies are therefore needed to elucidate the mechanisms by which biologic sex and gender are related to risk for PRTS and other poor health outcomes during the pandemic among older adults.

Pre-COVID anxiety symptoms also significantly predicted PRTS status after onset of the pandemic, with about 1 standard deviation (SD) difference between the groups in terms of pre-pandemic anxiety symptoms (+0.5 SD for PRTS+ and −0.5 SD for PRTS−, relative to the population norm), aligning with other reports that pre-pandemic anxiety is associated with greater PRTS in older adults (Maggi et al., 2021). An important implication for older adults with HTN and poorer pre-pandemic mental health (e.g. anxiety) may be unfavorable lifestyle changes (e.g. decreased physical activity, dietary choices), which have been reported elsewhere (Jääskeläinen et al., 2023), and may increase risk of CVD-related morbidity. This suggests that screening older adults with even sub-clinical anxiety symptoms for trauma-related symptoms during the pandemic is warranted.

Factors related to cardiovascular health, including anti-HTN medications and FRS scores, were not associated with PRTS status during the pandemic, nor were pre-COVID vascular and inflammatory biomarker levels. It is possible, however, that our study was underpowered to detect smaller effects that these factors may have exerted on PRTS risk. For instance, at the proportion of PRTS+ participants observed in the present study (i.e. 42%), a moderately sized effect (*d* = 0.30) detectable at 80% power would require ~150 PRTS+ individuals in the sample. Larger sample sizes may therefore be needed to detect group differences. It may also be the case that a true effect does not exist. While the literature supports bidirectional relationships between cardiovascular health and PTSD, it is unknown whether the physiologic or behavioral mechanisms at play would be applicable to sub-clinical traumatic stress symptoms, such as PRTS, which is the focus of the current investigation.

Our study had a handful of limitations. First, study participants were older adults with HTN who had previously enrolled in a clinical trial aimed at promoting health behaviors, which could introduce a selection bias. It is also notable that the participants who did not respond to the COVID-19 survey were older, less likely to be married, and had lower pre-COVID MoCA scores, compared to those who responded to the COVID-19 survey. Participants in this follow-up study therefore represent a slightly younger group with better pre-pandemic cognitive status, potentially with more social support, compared the larger parent study cohort. The current study may therefore underestimate the true prevalence of PRTS in older adults generally. Participants were predominantly Caucasian and college-educated, and largely without pre-existing illnesses other than HTN. They were also recruited from a geographic area with lower rates of infection but stricter lockdown measures during the early phase of the pandemic compared to other parts of the United States and globally, and no participants reported a laboratory-confirmed COVID-19 diagnosis. PRTS groups were balanced regarding these factors, but this could nevertheless limit the generalizability of the findings.

Further, PRTS was assessed using a rapid, 4-item, self-report measure, which was modified to assess trauma symptoms related to the pandemic, and clinical interviews to adjudicate PTSD diagnosis were not performed. While participants were prompted to respond to PC-PTSD questions with the COVID-19 pandemic as the trauma or stressor of reference, it is unknown to what degree the pandemic would meet the threshold of a “criterion A” trauma for each participant (i.e. exposure to actual or threatened death or serious injury). Trauma-related symptoms were also not assessed prior to the pandemic, and so the extent to which trauma history or pre-existing trauma-related symptoms influenced PRTS status during the pandemic is unknown. We also used a cut-off score of ⩾1 on the PC-PTSD, while this measure was developed as a screening tool for PTSD in primary care settings, with a cut-off score ⩾ 3 being the established cut-off score with optimal sensitivity, specificity, and efficiency for a clinical diagnosis of PTSD (Prins et al., 2004). However, we aimed to capture any perceived traumatic stress during the pandemic, rather than only probable clinical diagnoses. Further, we conducted a sensitivity analysis, wherein we compared sociodemographic and health-related measures between PRTS groups using both a PC-PTSD cut-off score of ⩾1 and ⩾3. In both analyses, the PRTS+ group had worse perception of their general and mental health, and greater perceived impairment in IADLs, suggesting that even subclinical traumatic stress symptoms during the pandemic are associated with poorer overall health status, and further investigations of these associations are warranted.

Despite these limitations, our findings demonstrate that older adults with HTN reported PRTS at higher rates than pre-pandemic estimates for the general population. However, prevalence is not greater than that in other adult samples during the pandemic. Pre-COVID indices of cardiovascular health and vascular and inflammatory biomarkers did not significantly predict risk of PRTS during the pandemic in the current sample, but this warrants investigation in a larger sample. Female gender and pre-pandemic anxiety levels did predict PRTS, indicating that screening for PRTS during the pandemic in older women with HTN and pre-existing anxiety symptoms might be warranted. For older adults with HTN who do report PRTS during the pandemic, poorer self-reported general and mental health, along with more impairment in activities of daily living are of concern. Given the known associations among HTN, traumatic stress, and poorer cardiovascular and cognitive outcomes, longitudinal studies are urgently needed to better characterize the impact of PRTS during the pandemic on trajectories of aging in older adults, as are interventions to mitigate the traumatic-stress related burden of the pandemic in this vulnerable population.

## Research Data

sj-csv-2-hpq-10.1177_13591053231213305 – Supplemental material for Post-traumatic stress in older, community-dwelling adults with hypertension during the COVID-19 pandemic: An investigation of pre-pandemic sociodemographic, health, and vascular and inflammatory biomarker predictors Supplemental material, sj-csv-2-hpq-10.1177_13591053231213305 for Post-traumatic stress in older, community-dwelling adults with hypertension during the COVID-19 pandemic: An investigation of pre-pandemic sociodemographic, health, and vascular and inflammatory biomarker predictors by Emily A Troyer, Jordan N Kohn, Monica Feliz R Castillo, Judith D Lobo, Yaniel Ramirez Sanchez, Gavrila Ang, Anthony Cirilo, Juan Andrew Leal, Christopher Pruitt, Amanda L Walker, Kathleen L Wilson, Meredith A Pung, Laura S Redwine and Suzi Hong in Journal of Health Psychology

sj-docx-1-hpq-10.1177_13591053231213305 – Supplemental material for Post-traumatic stress in older, community-dwelling adults with hypertension during the COVID-19 pandemic: An investigation of pre-pandemic sociodemographic, health, and vascular and inflammatory biomarker predictors Supplemental material, sj-docx-1-hpq-10.1177_13591053231213305 for Post-traumatic stress in older, community-dwelling adults with hypertension during the COVID-19 pandemic: An investigation of pre-pandemic sociodemographic, health, and vascular and inflammatory biomarker predictors by Emily A Troyer, Jordan N Kohn, Monica Feliz R Castillo, Judith D Lobo, Yaniel Ramirez Sanchez, Gavrila Ang, Anthony Cirilo, Juan Andrew Leal, Christopher Pruitt, Amanda L Walker, Kathleen L Wilson, Meredith A Pung, Laura S Redwine and Suzi Hong in Journal of Health Psychology

sj-pdf-3-hpq-10.1177_13591053231213305 – Supplemental material for Post-traumatic stress in older, community-dwelling adults with hypertension during the COVID-19 pandemic: An investigation of pre-pandemic sociodemographic, health, and vascular and inflammatory biomarker predictors Supplemental material, sj-pdf-3-hpq-10.1177_13591053231213305 for Post-traumatic stress in older, community-dwelling adults with hypertension during the COVID-19 pandemic: An investigation of pre-pandemic sociodemographic, health, and vascular and inflammatory biomarker predictors by Emily A Troyer, Jordan N Kohn, Monica Feliz R Castillo, Judith D Lobo, Yaniel Ramirez Sanchez, Gavrila Ang, Anthony Cirilo, Juan Andrew Leal, Christopher Pruitt, Amanda L Walker, Kathleen L Wilson, Meredith A Pung, Laura S Redwine and Suzi Hong in Journal of Health Psychology

sj-pdf-4-hpq-10.1177_13591053231213305 – Supplemental material for Post-traumatic stress in older, community-dwelling adults with hypertension during the COVID-19 pandemic: An investigation of pre-pandemic sociodemographic, health, and vascular and inflammatory biomarker predictors Supplemental material, sj-pdf-4-hpq-10.1177_13591053231213305 for Post-traumatic stress in older, community-dwelling adults with hypertension during the COVID-19 pandemic: An investigation of pre-pandemic sociodemographic, health, and vascular and inflammatory biomarker predictors by Emily A Troyer, Jordan N Kohn, Monica Feliz R Castillo, Judith D Lobo, Yaniel Ramirez Sanchez, Gavrila Ang, Anthony Cirilo, Juan Andrew Leal, Christopher Pruitt, Amanda L Walker, Kathleen L Wilson, Meredith A Pung, Laura S Redwine and Suzi Hong in Journal of Health Psychology

sj-pdf-5-hpq-10.1177_13591053231213305 – Supplemental material for Post-traumatic stress in older, community-dwelling adults with hypertension during the COVID-19 pandemic: An investigation of pre-pandemic sociodemographic, health, and vascular and inflammatory biomarker predictors Supplemental material, sj-pdf-5-hpq-10.1177_13591053231213305 for Post-traumatic stress in older, community-dwelling adults with hypertension during the COVID-19 pandemic: An investigation of pre-pandemic sociodemographic, health, and vascular and inflammatory biomarker predictors by Emily A Troyer, Jordan N Kohn, Monica Feliz R Castillo, Judith D Lobo, Yaniel Ramirez Sanchez, Gavrila Ang, Anthony Cirilo, Juan Andrew Leal, Christopher Pruitt, Amanda L Walker, Kathleen L Wilson, Meredith A Pung, Laura S Redwine and Suzi Hong in Journal of Health Psychology This article is distributed under the terms of the Creative Commons Attribution 4.0 License ( http://www.creativecommons.org/licenses/by/4.0/) which permits any use, reproduction and distribution of the work without further permission provided the original work is attributed as specified on the SAGE and Open Access pages ( https://us.sagepub.com/en-us/nam/open-access-at-sage).
