# Supplementary material for: Post-traumatic stress in older, community-dwelling adults with hypertension during the COVID-19 pandemic: An investigation of pre-pandemic sociodemographic, health, and vascular and inflammatory biomarker predictors
Source: J Health Psychol. 2023 Dec 13;29(6):552–66. doi: 10.1177/13591053231213305 (PMC11075414; doi:10.1177/13591053231213305)
Supplement: sj-docx-1-hpq-10.1177_13591053231213305 – Supplemental material for Post-traumatic stress in older, community-dwelling adults with hypertension during the COVID-19 pandemic: An investigation of pre-pandemic sociodemographic, health, and vascular and inflammatory biomarker predictors [file sj-docx-1-hpq-10.1177_13591053231213305.docx]

**Supplementary Materials**

**Supplementary Table 1**

*Pre-COVID Sociodemographic Characteristics for Parent Study Participants Who Responded to the COVID-19 Assessment Versus those Who Did Not*

|  | **Respondents (*n*=93)** | **Non-Respondents (*n*=40)** | **Test statistic,**  **[95% CI]** |
| --- | --- | --- | --- |
| Age: mean (SD) | 71.7 (6.94) | 74.9 (8.83) | *t*_61_ = 2.01, [0.013, 6.30] * |
| Gender: % female | 63/93 (67.7) | 30/39 (77.0) | *χ^2^* = 0.715 |
| Race: % Caucasian | 72/88 (81.8) | 35/39 (89.7) | *χ^2^* = 0.299 |
| Marital status: % married | 37/93 (39.8) | 6/40 (15.0) | *χ^2^* = 6.76 ** |
| First language: % English | 81/93 (87.1) | 33/40 (82.5) | *χ^2^* = 0.180 |
| Education: % some college or greater | 77/92 (83.7) | 34/37 (91.9) | *OR* = 0.455, [0.079, 1.76] |
| Study arm: % assigned to Tai Chi | 45/93 (48.4) | 24/40 (60.0) | *χ^2^* = 1.08 |

*Note.* CI = confidence interval. SD = standard deviation. *χ^2^* = Chi-squared. *OR* = odds ratio (based on Fisher’s Exact Test). **p*< 0.05, ***p*< 0.01, ****p*< 0.001.

**Supplementary Table 2**

*Pre-COVID General, Mental, Cognitive, and Cardiovascular Health Measures for Parent Study Participants Who Responded to the COVID-19 Assessment Versus those Who Did Not*

|  | **Respondents (*n*=93)** | **Non-Respondents (*n*=40)** | **Test statistic,**  **[95% CI]** |
| --- | --- | --- | --- |
| Smoking: % using tobacco | 0/91 (0.00) | 1/37 (2.70) | *OR* = 0.00,  [0.00, 15.9] |
| Cannabis: % any cannabis use | 9/91 (9.89) | 1/37 (2.70) | *OR* = 3.92,  [0.510, 178] |
| Alcohol: % any alcohol use | 54/90 (60.0) | 25/37 (67.6) | *χ^2^* = 0.357 |
| SF-20 social functioning: mean (SD) | 90.7 (20.6) | 82.2 (28.6) | *r_rb_* = -0.15,  [-0.36, 0.07] |
| SF-20 pain: mean (SD) | 67.3 (26.2) | 65.7 (23.1) | *r_rb_* = -0.04,  [-0.26, 0.19] |
| SF-20 health perception: mean (SD) | 67.2 (15.3) | 66.0 (18.8) | *r_rb_* = 0.07,  [-0.15, 0.29] |
| SF-20 role functioning: mean (SD) | 79.3 (36.0) | 66.7 (43.5) | *r_rb_* = -0.14,  [-0.35, 0.08] |
| SF-20 physical functioning: mean (SD) | 74.3 (26.5) | 68.4 (30.6) | *r_rb_* = -0.09,  [-0.31, 0.13] |
| PROMIS-SD: mean (SD) | 48.0 (10.0) | 48.5 (12.1) | *t*_55_ = 0.211,  [-4.09, 5.05] |
| SF-20 mental health: mean (SD) | 82.7 (15.8) | 79.3 (15.3) | *r_rb_* = -0,17,  [-0.38, 0.05] |
| BDI-II: mean (SD) | 5.30 (5.63) | 6.23 (6.93) | *r_rb_* = 0.00,  [-0.22, 0.22] |
| PROMIS-A: mean (SD) | 50.8 (8.41) | 52.7 (8.66) | *t*_63_ = 1.10,  [-1.52, 5.24] |
| CD-RISC-10: mean (SD) | 33.6 (5.80) | 32.7 (5.76) | *r_rb_* = -0.10,  [-0.31, 0.13] |
| GQ-6: mean (SD) | 36.4 (4.61) | 35.9 (4.97) | *r_rb_* = -0.06,  [-0.28, 0.16] |
| MoCA: mean (SD) | 26.3 (2.43) | 24.7 (3.97) | *r_rb_* = -0.24,  [-0.02, -0.44] * |
| SBP: mean (SD) | 132.2 (18.3) | 139.7 (19.6) | *t*_63_ = 2.00,  [-0.002, 15.0] |
| DBP: mean (SD) | 68.0 (9.50) | 71.9 (11.3) | *t*_57_ = 1.83,  [-0.360, 8.23] |
| Anti-HTN medications: mean (SD) | 1.34 (1.19) | 1.33 (1.39) | *r_rb_* = -0.04,  [-0.26, 0.18] |
| CRP: mean (SD), median | 3.70 (4.67), 1.89 | 4.48 (4.73), 2.60 | *r_rb_* = 0.15,  [-0.08, 0.36] |
| CRP >/= 3 pg/mL: proportion (%) | 18/80 (22.5) | 12/36 (33.3) | *χ^2^* = 1.01 |

*Note.* CI = confidence interval. SD = standard deviation. SF-20 = 20-Item Short Form Health Survey. PROMIS = Patient-Reported Outcomes Measurement Information System. PROMIS-SD = PROMIS Sleep Disturbance, Adult Short Form 8a. BDI-II = Beck Depression Inventory, Second Edition. PROMIS-A = PROMIS Anxiety, Adult Short Form 8a. CD-RISC-10 = 10-Item Connor-Davidson Resilience Scale. GQ-6 = Gratitude Questionnaire 6-Item Form. MoCA = Montreal Cognitive Assessment, Version 7. SBP = systolic blood pressure. DBP = diastolic blood pressure. HTN = hypertension. CRP = C-reactive protein. pg/mL = picograms per millileter. OR = odds ratio (based on Fisher’s Exact Test). *χ^2^* = Chi-squared. *r_rb_* = rank biserial correlation coefficient. **p*< 0.05, ***p*< 0.01, ****p*< 0.001.

**Supplementary Table 3**

*Time Since Pre-COVID Assessment and Start of Pandemic Lockdown to Completion of COVID-19 Survey, by PRTS Group*

|  | **PRTS+ (*n*=40)** | **PRTS- (*n*=55)** | **Test statistic,**  **[95% CI]** |
| --- | --- | --- | --- |
| Weeks elapsed between pre-COVID and COVID-19 assessments: mean (SD) | 128.1 (46.2) | 118.1 (40.2) | *t*_77_ = 1.09,  [8.16, 28.1] |
| Days elapsed from start of CA lockdown (March 19, 2020) to COVID-19 assessment: mean (SD) | 142.7 (36.0) | 125.2 (32.8) | *χ^2^* = 5.64^*^ |

*Note.* PRTS = pandemic-related traumatic stress symptoms. CI = confidence interval. SD = standard deviation. CA = California. *χ^2^* = Chi-squared. **p*< 0.05, ***p*< 0.01, ****p*< 0.001.

**Supplementary Table 4**

*COVID-19 Related Stressors Reported on the CRISIS Questionnaire Based on Self-Reported PRTS at the COVID-19 Assessment*

|  | **PRTS+**  **(*n*=40)** | **PRTS-**  **(*n*=55)** | **Test Statistic,**  **[95% CI]** |
| --- | --- | --- | --- |
| Living alone (%) | 14/40 (35.0) | 27/55 (49.1) | *χ^2^* = 1.34 |
| Past 2-week exposure to suspected COVID (%) | 0/40 (0.00) | 2/55 (3.64) | *OR* = 0.00,  [0.00, 7.32] |
| Personally suspected of having COVID (%) | 4/39 (10.3) | 1/54 (1.85) | *OR* = 0.168,  [0.003, 1.79] |
| Recent COVID-like symptoms (%) | 8/40 (20.0) | 4/55 (7.27) | *OR* = 3.15,  [0.77, 15.5] |
| Living with someone who has had COVID (%) | 1/39 (2.56) | 1/54 (1.85) | *OR* = 1.39,  [0.02, 111.4] |
| Someone close medically/financially impacted (%) | 12/40 (30.0) | 13/55 (23.6) | *χ^2^* = 0.211 |
| No longer working because of COVID (%) | 5/40 (12.5) | 6/53 (11.3) | *χ^2^* = 0.002 |
| Financial stress related to COVID (%) | 18/40 (45.0) | 21/55 (38.2) | *χ^2^* = 0.208 |
| Concerned about housing due to COVID (%) | 18/40 (45.0) | 16/55 (29.1) | *χ^2^* = 1.91 |
| Concerned about affording food due to COVID (%) | 3/40 (7.50) | 2/55 (3.64) | *OR* = 2.13,  [0.23, 26.7] |
| Difficulty affording basics due to COVID (%) | 7/40 (17.5) | 4/55 (7.27) | *OR* = 0.374,  [0.074, 1.61] |
| Loss of form(s) of support due to COVID (%) | 22/40 (55.0) | 30/54 (55.6) | *χ^2^* = 0.00 |

*Note.* PRTS = pandemic-related traumatic stress symptoms. *χ^2^* = Chi-squared. CI = confidence interval. *OR* = odds ratio (based on Fisher’s Exact Test). **p*< 0.05, ***p*< 0.01, ****p*< 0.001.

**Supplementary Table 5**

*Sociodemographic Characteristics at the Pre-COVID Assessment by PRTS ^#^ Group.*

|  | **PRTS+ (*n*=7)** | **PRTS- (*n*=88)** | **Test statistic,**  **[95% CI]** |
| --- | --- | --- | --- |
| Age: mean (SD) | 74.9 (10.4) | 71.3 (6.54) | *t*_6.38_ = -0.91,  [-13.3, 6.03] |
| Gender: % female | 7/7 (100) | 57/88 (64.8) | *OR =* inf |
| Race: % Caucasian | 4/6 (66.7) | 70/84 (83.3) | *OR =* 0.405 |
| Marital status: % married | 4/7 (66.7) | 35/88 (39.8) | *OR =* 2.00 |
| First language: % English | 7/7 (100) | 76/88 (86.4) | *OR =* inf |
| Education: % some college or greater | 7/7 (100) | 72/87 (82.8) | *OR =* inf |
| Study arm: % assigned to Tai Chi | 3/7 (42.9) | 42/88 (47.7) | *OR =* 0.823 |

*Note.* PRTS = pandemic-related traumatic stress symptoms. ^#^ PRTS groups defined using PC-PTSD cut-off score of 3. CI = confidence interval. SD = standard deviation. *χ^2^* = Chi-squared. **p*< 0.05, ***p*< 0.01, ****p*< 0.001.

**Supplementary Table 6**

*General, Mental, and Cognitive Health Self-Report Measures at the COVID-19 Assessment Based on Self-Reported PRTS ^#^*

|  | **PRTS+ (*n*=7)** | **PRTS- (*n*=88)** | **Test statistic, [95% CI]** |
| --- | --- | --- | --- |
| Smoking: % using tobacco | 0/7 (0.00) | 1/87 (1.15) | *OR* = 0.00,  [0.00, 481] |
| Cannabis: % any cannabis use | 0/7 (0.00) | 4/88 (4.55) | *OR* = 0.00,  [0.00, 21.4] |
| Alcohol: % any alcohol use | 4/7 (57.1) | 46/88 (52.3) | *OR* = 1.21,  [0.193, 8.78] |
| SF-20 social functioning: mean (SD) | 62.9 (31.5) | 84.3 (29.5) | *r_rb_* = 0.42,  [0.01, 0.72] |
| SF-20 pain: mean (SD) | 51.4 (22.7) | 62.3 (24.0) | *r_rb_* = 0.25,  [-0.19, 0.61] |
| SF-20 health perception: mean (SD) | 33.8 (19.0) | 56.3 (19.8) | *r_rb_* = 0.61,  [0.26, 0.82] |
| SF-20 role functioning: mean (SD) | 50.0 (50.0) | 76.7 (39.2) | *r_rb_* = 0.30,  [-0.13, 0.64] |
| SF-20 physical functioning: mean (SD) | 51.2 (33.1) | 71.2 (28.1) | *r_rb_* = 0.38,  [-0.15, 0.69] |
| PROMIS-SD: mean (SD) | 29.33 (7.63) | 16.11 (5.19) | *t_5.32_* = -4.18,  [-21.2, -5.23] ^**^ |
| SBQ: mean (SD) | 65.3 (12.7) | 66.2 (24.3) | *t*_9.99_ = 0.17,  [-11.2, 13.0] |
| SF-20 mental health: mean (SD) | 56 (14.1) | 81.8 (15.3) | *r_rb_* = 0.79,  [0.55, 0.91] |
| BDI-II: mean (SD) | 16.3 (5.79) | 5.97 (5.21) | *r_rb_* = 0.82,  [0.61, 0.92] |
| PROMIS-A: mean (SD) | 21.1 (6.09) | 13.3 (4.98) | *t*_6.65_ = -3.32,  [-13.5, -2.21] ^*^ |
| ULS-8: mean (SD) | 60.7 (19.8) | 45.4 (14.2) | *t*_6.50_ = -2.00,  [-33.6, 3.10] |
| CD-RISC-10: mean (SD) | 23.7 (7.89) | 30.1 (6.98) | *r_rb_* = 0.47,  [0.06, 0.74] |
| SCS-SF: mean (SD) | 34.6 (9.07) | 45.6 (8.30) | *r_rb_* = 0.61,  [0.25, 0.82] |
| GQ-6: mean (SD) | 33.6 (7.02) | 36.5 (4.99) | *r_rb_* = 0.24,  [-0.20, 0.60] |
| FAQ: mean (SD) | 4.00 (3.96) | 1.52 (2.71) | *r_rb_* = -0.52  [-0.77, -0.13] |

*Note.* PRTS = pandemic-related traumatic stress symptoms. ^#^ PRTS groups defined using PC-PTSD cut-off score of 3. CI = confidence interval. SF-20 = 20-Item Short Form Health Survey. SD = standard deviation. PROMIS = Patient-Reported Outcomes Measurement Information System. PROMIS-SD = PROMIS Sleep Disturbance, Adult Short Form 8a. SBQ = Sedentary Behavior Questionnaire. BDI-II = Beck Depression Inventory, Second Edition. PROMIS-A = PROMIS Anxiety, Adult Short Form 8a. ULS-8 = 8-Item UCLA Loneliness Scale. CD-RISC-10 = 10-Item Connor-Davidson Resilience Scale. SCS-SF = Self-Compassion Scale – Short Form. GQ-6 = Gratitude Questionnaire 6-Item Form. FAQ = Functional Activities Questionnaire. *OR* = odds ratio (based on Fisher’s Exact Test). *χ^2^* = Chi-squared. *r_rb_* = rank biserial correlation coefficient. **p*< 0.05, ***p*< 0.01, ****p*< 0.001.

**Supplementary Figure 1**

*Correlation Matrix of Pre-COVID Inflammatory and Vascular Injury Biomarker Levels Demonstrating High Multicollinearity*

*Note.* SAA = serum amyloid A. CRP = C-reactive protein. PC1 = principal component 1. sVCAM-1 = soluble vascular adhesion molecule-1. sICAM-1 = soluble intercellular adhesion molecule-1. IL-6 = interleukin-6. TNF = tumor necrosis factor-α. * *p*<0.0025. ** *p*<0.00025.

**Supplementary Figure 2**

*Spearman’s Correlations among Pre-COVID General, Mental, Cognitive, and Cardiovascular Health Indices*

**
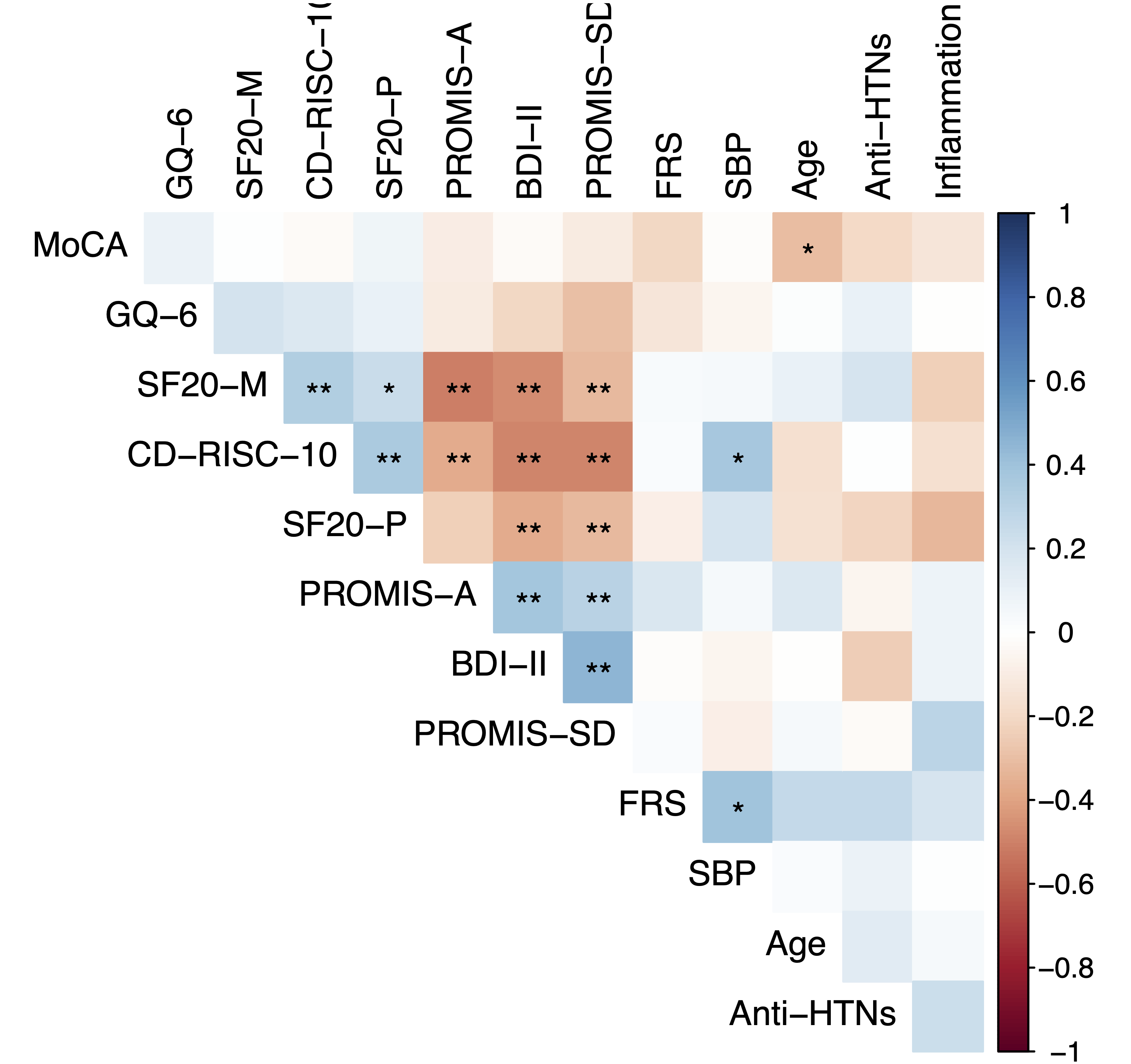
**

*Note.* MoCA = Montreal Cognitive Assessment, Version 7. GQ-6 = Gratitude Questionnaire 6-Item Form. SF20-M = 20-Item Short Form Health Survey, mental health subscale. CD-RISC-10 = 10-Item Connor-Davidson Resilience Scale. SF20-P = 20-Item Short Form Health Survey, physical functioning subscale. PROMIS = Patient-Reported Outcomes Measurement Information System. PROMIS-A = PROMIS Anxiety, Adult Short Form 8a. BDI-II = Beck Depression Inventory, Second Edition. PROMIS-SD = PROMIS Sleep Disturbance, Adult Short Form 8a. FRS = 10-year Framingham Risk Score. SBP = systolic blood pressure. Anti-HTNs = anti-hypertensive medications. * *p*<0.005. ***p*<0.0005.
