# Supplementary material for: Post-traumatic stress in older, community-dwelling adults with hypertension during the COVID-19 pandemic: An investigation of pre-pandemic sociodemographic, health, and vascular and inflammatory biomarker predictors
Source: J Health Psychol. 2023 Dec 13;29(6):552–66. doi: 10.1177/13591053231213305 (PMC11075414; doi:10.1177/13591053231213305)
Supplement: sj-pdf-3-hpq-10.1177_13591053231213305 – Supplemental material for Post-traumatic stress in older, community-dwelling adults with hypertension during the COVID-19 pandemic: An investigation of pre-pandemic sociodemographic, health, and vascular and inflammatory biomarker predictors [file sj-pdf-3-hpq-10.1177_13591053231213305.pdf]

Sociodemographic Variables			
**Variable Name**	**Question Text**	**Response Attributes**	**Visits**
subject_id	Not applicable. Subject ID variable entered by research staff.	Integer, assigned at study entry.<br>Min = 1, Max = 182	Available at baseline (pre-COVID) and COVID visits.
visit	Not applicable. Study visit variable entered by research staff.	Text.<br>BL = Study visit at baseline (pre-COVID)<br>COVID = Study visit during the COVID-19 pandemic	Available at baseline (pre-COVID) and COVID visits.
age	Not applicable. Age is calculated based on the difference between date of birth and date of study visit.	Numeric (continuous).<br>Min = 60.27, Max = 93.87<br>NA = missing	Available at baseline (pre-COVID) and COVID visits.
gender	Not applicable. Data entered by research staff based on participant response during semi-structured interview at V1.	Integer, single choice allowed.<br>1 = male<br>2 = female<br>NA = missing	Available at baseline (pre-COVID) and COVID visits.
race	Not applicable. Data entered by research staff based on participant response during semi-structured interview at V1.	Integer, single choice allowed.<br>1 = American Indian/Alaskan Native<br>2 = Asian<br>3 = Black or African American<br>4 = Native Hawaiian or Other Pacific Islander<br>5 = White<br>6 = More than one race<br>NA = missing	Available at baseline (pre-COVID) and COVID visits.
marital_status	Not applicable. Data entered by research staff based on participant response during semi-structured interview at V1.	Integer, single choice allowed.<br>1 = Single<br>2 = Married<br>3 = Living with a partner<br>4 = Separated<br>5 = Divorced<br>6 = Widowed<br>NA = missing	Available at baseline (pre-COVID) and COVID visits.

first_language	Not applicable. Data entered by research staff based on participant response during semi-structured interview at V1.	Integer, single choice allowed.<br>1 = English<br>2 = Spanish<br>3 = Other<br>NA = missing	Available at baseline (pre-COVID) and COVID visits.
ses1	Please indicate the highest level of education YOU have earned.<br>1. Less than 7 complete years of school<br>2. Junior high school (completed 7th, 8th, or 9th grade)<br>3. Partial high school (completed 10th or 11th grade)<br>4. High school graduate<br>5. Partial college (at least 1 year, including Associates Degree)<br>6. 4-year college degree (e.g., BS. BA)<br>7. Graduate training (e.g., MA, JD, MD, PhD), including partial	Integer, single choice allowed.<br>Min = 1, Max = 7<br>NA = missing	Available at baseline (pre-COVID) and COVID visits.
Primary Outcome of Interest			
**Variable Name**	**Question Text**	**Response Attributes**	**Visits**
ptsd_raw_total	Not applicable. This is a raw score for the 4-Item Primary Care PTSD Screen (PC-PTSD).	Numeric (continuous).<br>Min = 0, Max = 4<br>NA = missing	Available at COVID visit only.

COVID-19 Pandemic-Related Stressors			
**Variable Name**	**Question Text**	**Response Attributes**	**Visits**
crisis_res	What describes your current place of residence most accurately?<br>1. Private home in the general community<br>2. Residential unit in a building with shared entrance (e.g., apartment, condo)<br>3. Private home in a retirement community<br>4. Residential unit in a retirement building<br>5. Assisted living community or facility<br>6. Other, please specify:	Integer, single choice allowed.<br>Min = 1, Max = 6<br>NA = missing	Available at COVID visit only.
crisis_exp_other	During the past two weeks, have you been exposed to someone likely to have Coronavirus/COVID-19?<br>0. No<br>1. Yes	Integer, single choice allowed.<br>Min = 0, Max = 1<br>NA = missing	Available at COVID visit only.
crisis_exp_self	Have you personally been suspected of having Coronavirus/COVID-19 infection?<br>1. Yes, I had a positive test<br>2. Yes, I had a medical diagnosis, but no test<br>3. Yes, I have had some possible symptoms, but no diagnosis by a doctor<br>4. No, I have not had any symptoms or signs	Integer, single choice allowed.<br>Min = 1, Max = 4<br>NA = missing	Available at COVID visit only.
crisis_symp	During the past two weeks, have you had any of the following symptoms (fever, cough, shortness of breath, sore throat, fatigue, loss of taste or smell, eye infection, or other)?<br>0. No<br>1. Yes	Integer, single choice allowed.<br>Min = 0, Max = 1<br>NA = missing	Available at COVID visit only.
crisis_exp_home	Has anyone in your family or living space been diagnosed with Coronavirus/COVID-19?<br>0. No<br>1. Yes	Integer, single choice allowed.<br>Min = 0, Max = 1<br>NA = missing	Available at COVID visit only.

crisis_exp_event	Since the beginning of California's stay-at-home order (March 19, 2020), have any of the following happened to anyone in your family or household because of Coronavirus/COVID-19 (fallen ill physically, hospitalized, put into self-quarantine with symptoms, put into self-quarantine without symptoms due to possible exposure, lost or been laid off from job, reduced ability to earn money, or passed away)?<br>0. No<br>1. Yes	Integer, single choice allowed.<br>Min = 0, Max = 1<br>NA = missing	Available at COVID visit only.
crisis_employment	If you had a job prior to Coronavirus/COVID-19, are you still working?<br>0. Not applicable<br>1. Yes<br>2. No	Integer, single choice allowed.<br>Min = 0, Max = 2<br>NA = missing	Available at COVID visit only.
crisis_finan_stress	To what degree have changes related to the Coronavirus/COVID-19 crisis created financial problems for you or your family?<br>1. Not at all<br>2. Slightly<br>3. Moderately<br>4. Very<br>5. Extremely	Integer, single choice allowed.<br>Min = 1, Max = 5<br>NA = missing	Available at COVID visit only.
crisis_res_stress	Since the beginning of the California's stay-at-home order (March 19, 2020), to what degree are you concerned about the stability of your living situation?<br>1. Not at all<br>2. Slightly<br>3. Moderately<br>4. Very<br>5. Extremely	Integer, single choice allowed.<br>Min = 1, Max = 5<br>NA = missing	Available at COVID visit only.

crisis_food_stress	Since the beginning of California's stay-at-home order (March 19, 2020), did you worry about whether your food would run out because of lack of money?<br>0. No<br>1. Yes	Integer, single choice allowed.<br>Min = 0, Max = 1<br>NA = missing	Available at COVID visit only.
crisis_ses	Since the beginning of California's stay-at-home order (March 19, 2020), how hard is it for you to pay for the very basics like food, housing, medical care, and heating?<br>1. Not at all hard<br>2. Somewhat hard<br>3. Very hard	Integer, single choice allowed.<br>Min = 1, Max = 3<br>NA = missing	Available at COVID visit only.
crisis_supp_loss	Were any of the following supports in place for you before the Coronavirus/COVID-19 crisis in your area, and also disrupted since the beginning of California's stay-at-home order (March 19, 2020): resource room, tutoring, mentoring programs, volunteer programs, psychotherapy, psychiatric care, occupational therapy, physical therapy, speech/language therapy, sporting activities, medical care for chronic illnesses, or other?<br>0. No<br>1. Yes	Integer, single choice allowed.<br>Min = 0, Max = 1<br>NA = missing	Available at COVID visit only.
General Health and Behavior			
**Variable Name**	**Question Text**	**Response Attributes**	**Visits**
current_smoker	Are you currently using any products containing nicotine (e.g., smokeless tobacco, cigarettes, e-cigarettes, vaping, cigar, pipe, nicotine patch, nicotine gum, etc.)?<br>0. No<br>1. Yes	Integer, single choice allowed.<br>Min = 0, Max = 1<br>NA = missing	Available at baseline (pre-COVID) and COVID visits.

mj_use	Are you currently using marijuana?<br>0. No<br>1. Yes<br>2. In the past	Integer, single choice allowed.<br>Min = 0, Max = 2<br>NA = missing	Available at baseline (pre-COVID) and COVID visits.
current_alcohol	Do you drink alcohol?<br>0. No<br>1. Yes<br>2. In the past	Integer, single choice allowed.<br>Min = 0, Max = 2<br>NA = missing	Available at baseline (pre-COVID) and COVID visits.
sf20_social	Not applicable. This is a weighted score for the social functioning subscale of the 20-Item Short Form Health Survey (SF-20).	Numeric (continuous).<br>Min = 0, Max = 100<br>NA = missing	Available at baseline (pre-COVID) and COVID visits.
sf20_pain	Not applicable. This is a weighted score for the pain subscale of the 20-Item Short Form Health Survey (SF-20).	Numeric (continuous).<br>Min = 1, Max = 120<br>NA = missing	Available at baseline (pre-COVID) and COVID visits.
sf20_perceptions	Not applicable. This is a weighted score for the health perceptions subscale of the 20-Item Short Form Health Survey (SF-20).	Numeric (continuous).<br>Min = 3.96, Max = 87<br>NA = missing	Available at baseline (pre-COVID) and COVID visits.
sf20_role	Not applicable. This is a weighted score for the role functioning subscale of the 20-Item Short Form Health Survey (SF-20).	Numeric (continuous).<br>Min = 0, Max = 100<br>NA = missing	Available at baseline (pre-COVID) and COVID visits.
sf20_physical	Not applicable. This is a weighted score for the physical functioning subscale of the 20-Item Short Form Health Survey (SF-20).	Numeric (continuous).<br>Min = 0, Max = 100<br>NA = missing	Available at baseline (pre-COVID) and COVID visits.
sleepdisturb_total_weighted	Not applicable. This is a weighted score for the Patient Reported Outcomes Measurement Information System (PROMIS) Sleep Disturbance scale, Adult Short Form 8a.	Numeric (continuous).<br>Min = 8, Max = 38.67<br>NA = missing	Available at baseline (pre-COVID) and COVID visits.

sed_weighted_total	Not applicable. This is a weighted score for the Sedentary Behavior Questionnaire (SBQ).	Numeric (continuous).<br>Min = 12.25, Max = 138.25<br>NA = missing	Available at baseline (pre-COVID) and COVID visits.
Mental Health			
**Variable Name**	**Question Text**	**Response Attributes**	**Visits**
sf20_mental	Not applicable. This is a weighted score for the mental health subscale of the 20-Item Short Form Health Survey (SF-20).	Numeric (continuous).<br>Min = 12, Max = 100<br>NA = missing	Available at baseline (pre-COVID) and COVID visits.
bdi_weighted_total	Not applicable. This is a weighted score for the Beck Depression Inventory, Second Edition (BDI-II).	Numeric (continuous).<br>Min = 0, Max = 40<br>NA = missing	Available at baseline (pre-COVID) and COVID visits.
anxiety_total_weighted	Not applicable. This is a weighted score for the Patient Reported Outcomes Measurement Information System (PROMIS) Anxiety scale, Adult Short Form 8a.	Numeric (continuous).<br>Min = 8, Max = 38<br>NA = missing	Available at baseline (pre-COVID) and COVID visits.
cdrisc_total_weighted	Not applicable. This is a weighted score for the 10-Item Connor-Davidson Resilience Scale (CD-RISC-10).	Numeric (continuous).<br>Min = 10, Max = 40<br>NA = missing	Available at baseline (pre-COVID) and COVID visits.
gq6_total_weighted	Not applicable. This is a weighted score for the Gratitude Questionnaire 6-Item Form (GQ-6).	Numeric (continuous).<br>Min = 18, Max = 42<br>NA = missing	Available at baseline (pre-COVID) and COVID visits.
uls_total_weighted	Not applicable. This is a weighted score for the 8-Item UCLA Loneliness Scale (ULS-8).	Numeric (continuous).<br>Min = 8, Max = 27<br>NA = missing	Available at COVID visit only.

neff_total_weighted	Not applicable. This is a weighted score for the Self Compassion Scale – Short Form (SCS-SF).	Numeric (continuous).<br>Min = 24, Max = 60<br>NA = missing	Available at COVID visit only.
Cognitive Health			
**Variable Name**	**Question Text**	**Response Attributes**	**Visits**
moca_total		Numeric (continuous).<br>Min = 11, Max = 30<br>NA = missing	Available at baseline (pre-COVID) visit only.
faq_total_weighted	Not applicable. This is a weighted score for the Functional Activities Questionnaire (FAQ).	Numeric (continuous).<br>Min = 0, Max = 12<br>NA = missing	Available at COVID visit only.
Cardiovascular Health			
**Variable Name**	**Question Text**	**Response Attributes**	**Visits**
num_anti_htns	Not applicable. This is a sum of unique anti-hypertensive medication classes prescribed.	Numeric (continuous).<br>Min = 0, Max = 6<br>NA = missing	Available at baseline (pre-COVID) and COVID visits.
sbp_sitting_mean	Not applicable. This is a mean systolic blood pressure calculated based on 3 consecutive measurements using an automated oscillometric sphygmomanometer.	Numeric (continuous).<br>Min = 88, Max = 185<br>NA = missing	Available at baseline (pre-COVID) visit only.
dbp_sitting_mean	Not applicable. This is a mean diastolic blood pressure calculated based on 3 consecutive measurements using an automated oscillometric sphygmomanometer.	Numeric (continuous).<br>Min = 46, Max = 95<br>NA = missing	Available at baseline (pre-COVID) visit only.
frs_10y	Not applicable. This is a Framingham Risk Score for each participant, calculated based on biologic sex, age, total cholesterol, HDL cholesterol, cigarette smoking status, and systolic blood pressure.	Numeric (continuous).<br>Min = 0.04, Max = 0.67<br>NA = missing	Available at baseline (pre-COVID) visit only.

Inflammatory and Vascular Injury Biomarkers			
**Variable Name**	**Question Text**	**Response Attributes**	**Visits**
CRP_MSD	Not applicable. This is the concentration of plasma C-reactive protein (CRP) measured via immunoassay.	Numeric (continuous).<br>Min = 0, Max = 26.07<br>NA = missing	Available at baseline (pre-COVID) visit only.
SAA_MSD	Not applicable. This is the concentration of plasma serum amyloid A (SAA) measured via immunoassay.	Numeric (continuous).<br>Min = 0.43, Max = 39.76<br>NA = missing	Available at baseline (pre-COVID) visit only.
sVCAM1_MSD	Not applicable. This is the concentration of plasma soluble vascular cell adhesion molecule-1 (sVCAM-1) measured via immunoassay.	Numeric (continuous).<br>Min = 0, Max = 1.76<br>NA = missing	Available at baseline (pre-COVID) visit only.
sICAM1_MSD	Not applicable. This is the concentration of plasma soluble intercellular adhesion molecule-1 (sICAM-1) measured via immunoassay.	Numeric (continuous).<br>Min = 0, Max = 1.23<br>NA = missing	Available at baseline (pre-COVID) visit only.
il6	Not applicable. This is the concentration of plasma interleukin-6 (IL-6) measured via immunoassay.	Numeric (continuous).<br>Min = 0.12, Max = 39.15<br>NA = missing	Available at baseline (pre-COVID) visit only.
tnf	Not applicable. This is the concentration of plasma tumor necrosis factor alpha (TNFα) measured via immunoassay.	Numeric (continuous).<br>Min = 0.46, Max = 277.41<br>NA = missing	Available at baseline (pre-COVID) visit only.
