# Supplementary material for: Post-traumatic stress in older, community-dwelling adults with hypertension during the COVID-19 pandemic: An investigation of pre-pandemic sociodemographic, health, and vascular and inflammatory biomarker predictors
Source: J Health Psychol. 2023 Dec 13;29(6):552–66. doi: 10.1177/13591053231213305 (PMC11075414; doi:10.1177/13591053231213305)
Supplement: sj-pdf-4-hpq-10.1177_13591053231213305 – Supplemental material for Post-traumatic stress in older, community-dwelling adults with hypertension during the COVID-19 pandemic: An investigation of pre-pandemic sociodemographic, health, and vascular and inflammatory biomarker predictors [file sj-pdf-4-hpq-10.1177_13591053231213305.pdf]

Statistical analyses were conducted in R (v.3.6.0). All variables were inspected for normality and outliers.

COVID-19 survey respondents were compared to non-respondents in terms of pre-pandemic sociodemographic variables and general, mental, cognitive, and cardiovascular health variables. Group comparisons were conducted using nonparametric, rank-biserial correlation coefficients ($r_{rb}$) for non-normal continuous variables. Welch's t-test for normally distributed continuous variables, and categorical variables were compared using Chi-squared ($\chi^2$) or Fisher's exact tests (see Supplementary Tables 1-2).

Prevalence of PTSS was calculated based on the proportion of participants with PC-PTSD scores >/= 3, and participants with PC-PTSD scores >/= 1 were classified as 'PTSS+'. PTSS+ and PTSS- groups were compared regarding sociodemographic, pandemic-related stressor, general health and behavior, mental health, and cognitive health variables. Comparisons were conducted using nonparametric, rank-biserial correlation coefficients ($r_{rb}$) for non-normal continuous variables. Welch's t-test for normally distributed continuous variables, and categorical variables were compared using Chi-squared ($\chi^2$) or Fisher's exact tests (see Tables 1-2 and Supplementary Table 3).

Pre-COVID predictors of PTSS group status during the COVID-19 pandemic included sociodemographic factors and general, mental, cognitive, and cardiovascular health variables, as well as inflammatory and vascular injury biomarkers. Correlation analyses were used to assess associations among inflammatory and vascular injury biomarker predictors (see Supplementary Figure 1) and among all pre-COVID predictors (see Supplementary Figure 2).

Dimensionality reduction by principal components analysis (PCA) was performed using *principal* in *psych* for the 6 inflammatory and vascular injury biomarkers due to their high

multicollinearity to extract component scores for each participant. Complete biomarker data were available for 80 of 95 (84%) of participants, and missing values were imputed using an iterative, regularized PCA algorithm in the *missMDA* package. Imputed biomarker data were scaled and standardized prior to PCA, for which the first component (PC1) accounted for 48% of the total variance and upon which all biomarkers loaded stably (standardized loadings ≥ 0.60), except for TNF-α (0.30). Varimax-rotated component scores from PC1 were used as the inflammatory and vascular injury biomarker index in logistic regression analysis, and correlations between PC1 and other variables were described. False discovery rate (FDR) correction was applied to biomarker correlations to control Type I error rate due to multiple comparisons.

Logistic regression was employed to assess pre-COVID predictors of PTSS group status during the COVID-19 pandemic. Missingness was <5% for all regression predictors, except Framingham Risk Score (FRS), which had ~20% missingness. Multiple imputation by chained equations (*mice*) using predictive mean matching with 100 imputations was implemented to account for missing data in logistic regression analyses. Binomial logistic regression was applied to each of the imputed datasets, and results were pooled to determine odds ratios (*ORs*) and 95% confidence intervals for each baseline variable in predicting 'PTSS+' status during the pandemic. Likelihood ratio tests were computed from pooled models using the *D3*-statistic (Meng & Rubin, 1992) to compare a reduced model containing age, gender, number of anti-HTN medications, FRS, and inflammatory and vascular injury biomarkers, to a full model that also contained depressive symptoms, anxiety, sleep disturbance, and MoCA scores (see Table 3).
