# Supplementary material for: Post-traumatic stress in older, community-dwelling adults with hypertension during the COVID-19 pandemic: An investigation of pre-pandemic sociodemographic, health, and vascular and inflammatory biomarker predictors
Source: J Health Psychol. 2023 Dec 13;29(6):552–66. doi: 10.1177/13591053231213305 (PMC11075414; doi:10.1177/13591053231213305)
Supplement: sj-pdf-5-hpq-10.1177_13591053231213305 – Supplemental material for Post-traumatic stress in older, community-dwelling adults with hypertension during the COVID-19 pandemic: An investigation of pre-pandemic sociodemographic, health, and vascular and inflammatory biomarker predictors [file sj-pdf-5-hpq-10.1177_13591053231213305.pdf]

The data for this empirical investigation have been made fully accessible in accordance with the *Journal of Health Psychology's* open publication model.

Please reference the following files:

1. Raw Data.csv – This is a comma separated values (CSV) file containing all data for this empirical investigation.
2. Data Dictionary.pdf – This is a portable document format (PDF) file containing a description of all variables from the data set.
3. Data Analyses.pdf – This is a PDF file containing a description of how all statistical analyses were conducted (in R, v.3.6.0). Replication can be conducted using the description of analyses in this file.
